# Genetic association of apolipoprotein E genotype with EEG alpha rhythm slowing and functional brain network alterations during normal aging

**DOI:** 10.3389/fnins.2022.931173

**Published:** 2022-08-01

**Authors:** Natalya V. Ponomareva, Tatiana V. Andreeva, Maria Protasova, Rodion N. Konovalov, Marina V. Krotenkova, Ekaterina P. Kolesnikova, Daria D. Malina, Elena V. Kanavets, Andrey A. Mitrofanov, Vitaly F. Fokin, Sergey N. Illarioshkin, Evgeny I. Rogaev

**Affiliations:** ^1^Research Center of Neurology, Moscow, Russia; ^2^Center for Genetics and Life Science, Sirius University of Science and Technology, Sochi, Russia; ^3^Vavilov Institute of General Genetics, Russian Academy of Sciences (RAS), Moscow, Russia; ^4^Research Center of Mental Health, Moscow, Russia; ^5^Brudnick Neuropsychiatric Research Institute (BNRI), University of Massachusetts Medical School, Worcester, MA, United States

**Keywords:** *APOE* genotype, alpha rhythm, functional MRI, functional connectivity, brain networks, aging, Alzheimer’s disease, genetic predisposition

## Abstract

The ε4 allele of the apolipoprotein E (*APOE*4+) genotype is a major genetic risk factor for Alzheimer’s disease (AD), but the mechanisms underlying its influence remain incompletely understood. The study aimed to investigate the possible effect of the *APOE* genotype on spontaneous electroencephalogram (EEG) alpha characteristics, resting-state functional MRI (fMRI) connectivity (rsFC) in large brain networks and the interrelation of alpha rhythm and rsFC characteristics in non-demented adults during aging. We examined the EEG alpha subband’s relative power, individual alpha peak frequency (IAPF), and fMRI rsFC in non-demented volunteers (age range 26–79 years) stratified by the *APOE* genotype. The presence of the *APOE4+* genotype was associated with lower IAPF and lower relative power of the 11–13 Hz alpha subbands. The age related decrease in EEG IAPF was more pronounced in the *APOE4+* carriers than in the *APOE4+* non-carriers (*APOE4*-). The *APOE4+* carriers had a stronger fMRI positive rsFC of the interhemispheric regions of the frontoparietal, lateral visual and salience networks than the *APOE4–* individuals. In contrast, the negative rsFC in the network between the left hippocampus and the right posterior parietal cortex was reduced in the *APOE4+* carriers compared to the non-carriers. Alpha rhythm slowing was associated with the dysfunction of hippocampal networks. Our results show that in adults without dementia *APOE4+* genotype is associated with alpha rhythm slowing and that this slowing is age-dependent. Our data suggest predominant alterations of inhibitory processes in large-scale brain network of non-demented *APOE4+* carriers. Moreover, dysfunction of large-scale hippocampal network can influence *APOE*-related alpha rhythm vulnerability.

## Introduction

Alzheimer’s disease (AD) is one of the most widespread neurodegenerative diseases and may contribute to 60-70% of dementia cases (Alzheimer’s Association, 2017). The greatest known risk factors for AD are genetic predisposition and aging. Early onset AD (EOAD) develops before the age of 65 years. The heritability EOAD is estimated around 92–100%. Mutations in the amyloid precursor protein (*APP*), presenilin-1 (*PRES-1*), and presenilin-2 (*PRES-2*) genes (Goate et al., 1991; Levy-Lahad et al., 1995; Rogaev et al., 1995; Sherrington et al., 1995) account for 5–10% of EOAD cases (Cuyvers and Sleegers, 2016).

A common polymorphism in the apolipoprotein E (*APOE*) genotype located on chromosome 19q13 has been established as the most prevalent genetic risk factor for late-onset AD in Caucasian ethnic groups, which include the Russian ethnic population used in this study (Saunders et al., 1993; Schmechel et al., 1993; Farrer et al., 1997; Rogaev, 1999). *APOE* is mainly expressed in astrocytes and other glial cells, but neurons also express *APOE* under stress (Fernandez et al., 2019). The glycoprotein APOE is involved in lipid transport, synaptogenesis, neural repair, neuroplasticity, neuroinflammation, and beta-amyloid (Aβ) clearance (Kim et al., 2014; Mahoney-Sanchez et al., 2016, reviews). *APOE* has three alleles (ε2, ε3, and ε4). The *APOE* ε4 genotype (*APOE4*+) is related to Aβ plaque and tau tangle accumulations in the brain, which are the cardinal neuropathological hallmarks of AD (Koutsodendris et al., 2022). *APOE4+* can contribute to AD by several Aβ-independent pathways. Through these pathways it can detrimentally affect neurogenesis, synaptic plasticity, white matter integrity, cerebral energy metabolism, neurovascular function and neuroinflammation (Kim et al., 2014).

Despite significant progress in AD research, there is no cure for AD. Because of this it is particularly important to find preclinical AD biomarkers and ways of preventing the disease (Rogaev et al., 1995; Illarioshkin et al., 2004; Furney et al., 2011; Dubois et al., 2014). According to the recently proposed diagnostic criteria of AD, the preclinical AD stage is characterized by the absence of clinical signs and symptoms of AD and the presence of at least one biomarker of Alzheimer’s pathology (decreased level of Aβ_42_, increased level of tau p-tau in cerebrospinal fluid or abnormal deposition of Aβ and tau in the brain that can be revealed by positron emission tomography) (Dubois et al., 2014).

Promising neurophysiological candidate biomarkers of preclinical AD have been suggested. electroencephalography (EEG) and magnetoencephalography (MEG) are non-invasive techniques that can be used to measure brain electromagnetic activity with a high temporal resolution of milliseconds. Compared to healthy age-matched controls, AD patients exhibit a slowing of the dominant EEG frequency, increased delta and theta power, and decreased alpha power (Rossini et al., 2007; Ponomareva et al., 2008; Lizio et al., 2011; van Straaten et al., 2014; Babiloni et al., 2018a). EEG of AD patients exhibits alterations of functional connectivity and of the complexity of EEG signal temporal dynamics, including changes of entropy measures (Hata et al., 2016; Núñez et al., 2019). In amnestic mild cognitive impairment (aMCI), which is in most cases a prodromal stage of AD, EEG characteristics are intermediate to those of normal subjects and AD patients (Babiloni et al., 2013; Lejko et al., 2020, review).

Resting-state alpha rhythm alterations represent one of the most significant changes related to AD development. Patients with AD and aMCI demonstrated a decrement of resting-state alpha rhythm power compared to healthy elderly subjects (Garcés et al., 2013; Babiloni et al., 2018a,b). In groups of individuals with subjective cognitive decline (SCD) and aMCI, alterations in alpha activity correlated with cognitive worsening have been detected (Puttaert et al., 2021). The alpha band power reduction is assumed to be related to synaptic damage which affects mostly cholinergic connections and results in impairment of the synchronization of higher-frequency activities (Lizio et al., 2011, review).

The heritability of EEG patterns has been shown to be in the range of 70–90% (van Beijsterveldt et al., 1996). Previous EEG and MEG studies demonstrated that these techniques can be used for the identification of endophenotypes, which are the basic heritable quantitative biological markers, characterizing the disease even at its preclinical stage. Research has indicated that an association exists between EEG characteristics and AD risk variants in the *APOE, CLU, PICALM*, *IL1RAP, UNC5C*, and *NAV2*, genes in AD and MCI patients and even in healthy adults (Jelic et al., 1997; Lehtovirta et al., 2000; Babiloni et al., 2006; Kramer et al., 2008; Ponomareva et al., 2008, 2012, 2017, 2020; Canuet et al., 2012; Lee et al., 2012; de Waal et al., 2013; Gutiérrez-de Pablo et al., 2020). However, our knowledge regarding the impact of AD risk variants on EEG parameters in healthy individuals across their lifespan remains incomplete.

Recently, an MEG study of healthy middle-aged and elderly adults demonstrated an association of the *APOE* genotype with individual alpha peak frequency (IAPF) (de Frutos-Lucas et al., 2018, 2020). IAPF is defined as the frequency of the strongest alpha oscillation observed by EEG during rest (Mierau et al., 2017). It is prominent over the occipital and parietal brain regions when the eyes are closed (Ramsay et al., 2021). In healthy subjects IAPF declines after 40 years of age (Scally et al., 2018). IAPF is lower in patients with MCI and AD and its decline correlates with cognitive worsening (Richard Clark et al., 2004). It is not clear whether *APOE*-related IAPF differences are age-dependent and whether *APOE*-related IAPF differences can be detected in healthy young adults.

Resting-state functional magnetic resonance tomography (fMRI) is another powerful technique that is able to reveal abnormalities of functional brain networks in AD, MCI, and non-demented subjects with a genetic predisposition to AD (Dennis and Thompson, 2014, review; Pietzuch et al., 2019, review; Foo et al., 2020, review; Watanabe et al., 2021, review). fMRI enables indirect assessment of neural activity by detecting changes in the blood oxygenation level dependent (BOLD) signals, that occur in response to neural activity through neurovascular coupling. In resting-state fMRI studies brain networks are defined by their patterns of spontaneous fluctuations in BOLD signals. fMRI studies have provided evidence that even though AD can be characterized as a disconnection syndrome (Brier et al., 2014a; Puttaert et al., 2020), there are phases of hyper- and hypoconnectivity during AD development, with the former phase preceding the latter (Schultz et al., 2017). The *APOE4+* genotype was previously shown to be linked with abnormal connectivity in brain networks of patients with AD and of clinically healthy subjects (Filippini et al., 2011; Damoiseaux et al., 2012; Reinvang et al., 2013, review; Dennis and Thompson, 2014, review; Foo et al., 2020; Henson et al., 2020; Wang et al., 2020; Pietzuch et al., 2021; Kang et al., 2022). Previous studies using resting state EEG and fMRI in healthy adults showed positive associations of alpha power with BOLD signal in the thalamus, and more heterogeneous associations in cortical regions (Brueggen et al., 2017; Marino et al., 2019). The link between the alterations in the fMRI-derived resting-state brain network and alpha rhythm slowing in *APOE4+* non-demented carriers has not yet been investigated. Moreover, the extent to which specific neurophysiological alterations in *APOE4+* carriers can be attributed to the course of aging remains underexplored.

The study aimed to determine whether *APOE* genotype is associated with alpha rhythm characteristics of spontaneous EEG in adults without dementia during aging. In addition, we explored the association of the *APOE* genotype and fMRI-derived rsFC of brain networks in these individuals. We also aimed to clarify the interrelation between the explored alpha rhythm and fMRI-derived rsFC characteristics. Complementary fMRI and EEG methods were applied to unveil the causes of alpha rhythm alterations in *APOE4+* carriers.

In our research, we examined the same individuals with both rsFC fMRI and EEG alpha characteristics. Such an approach maximizes the advantages of these methods for exploring *APOE*-related neurophysiological alterations in non-demented individuals. In the resting state of healthy subjects where the participant state and behavior are relatively stable, separate EEG and fMRI recordings across experimental sessions are sufficient (Scrivener, 2021).

## Materials and methods

### Participants

The enrolled cohort included 137 volunteers without dementia (45 men and 92 women, age range 26–79 years).

The subjects were of Russian descent and were from Moscow and the surrounding region. The participants underwent a neurological examination and cognitive screening. The recruited subjects were free of dementia and other medical, psychiatric, and neurological conditions. The exclusion criteria included a history of neurological and psychiatric diseases, any type of memory impairment, signs of clinical depression or anxiety, physical brain injury or other medical conditions (e.g., hypertension, diabetes, cardiac disease, or thyroid disease) or a personal history of drug or alcohol addiction.

The subjects were evaluated with the mini-mental state examination (MMSE) (Folstein et al., 1975) and clinical dementia rating (CDR) scale (Hughes et al., 1982). Only subjects with MMSE scores of 28 or higher and CDR scores of 0 were included in the study. All subjects were right-handed. All individuals also underwent a neuropsychological battery that included the following tests: the controlled oral word association test (COWAT) (Benton and Hamsher, 1989), Luria memory words test (LMWT) (Luria, 1966), serial sevens subtraction test (SST) (Milstein et al., 1972). COWAT examine**s** verbal fluency (i.e., the ability to produce words orally within a fixed time span according to phonemic constraints), task relies on circuits that control aspects of executive function (attention, initiation, and retrieval processes) and working memory (Benton and Hamsher, 1989). LWMT was applied to assess verbal memory deficits.

Written informed consent was obtained from all the participants. The experimental protocol for this study was approved by the local Ethics Committee. *APOE* genotyping was performed for all participants.

All subjects were divided into subgroups according to *APOE* polymorphism. The *APOE4+* subgroups consisted of subjects with one or more e4 *APOE* alleles, and the *APOE4–* subgroups included the subjects without any ε4 allele. Eighty-six of the *APOE4+* non-carriers had the ε3/ε3 genotype, five had the ε2/ε3 genotype; forty-four of the 46 *APOE4+* carriers had the ε4/ε3 genotype, two had the ε4/ε4 genotype. Each group was further subdivided into cohorts of those younger than and those older than 50 years of age. Table 1 shows the demographic and cognitive characteristics of the participants.

**TABLE 1:** Demographic and psychometric characteristics of participants.

	Younger than 50			Older than 49			All		
	*APOE4–*	*APOE4+*	*p*	*APOE4–*	*APOE4+*	*p*	*APOE4–*	*APOE4+*	*p*
*N*	38	16		53	30		91	46	
Age, years (SE)	38.8 (1.3)	39.5 (1.7)	0.8	59.1 (0.9)	59.8 (1.4)	0.7	50.6 (1.3)	52.7 (1.8)	0.3
Sex m/w	16/22	4/12	0.2	18/35	7/23	0.5	34/57	11/35	0.1
Education, years	15.0 (0.2)	15.0 (0.4)	0.9	15.1 (0.1)	15.0 (0.1)	0.6	15.1 (0.1)	15.0 (0.16)	0.8
MMSE (SE)	29.9 (0.14)	30.0 (0.1)	0.6	29.6 (0.2)	29.5 (0.3)	0.8	29.7 (0.1)	29.6 (O.3)	0.9
LMWT (SE)	6.0 (0.2)	5.9 (0.4)	0.8	5.4 (0.2)	5.2 (0.3)	0.6	5.6 (0.2)	5.4 (0.2)	0.4
COWAT (SE)	54.2 (2.5)	52.7 (3.2)	0.7	47.1 (1.6)	47.1 (1.8)	1.0	50.0 (1.4)	48.9 (1.6)	0.7
SST (SE)	1.0 (0.2)	0.8 (0.3)	0.6	1.0 (0.15)	0.6 (0.15)	0.6	1.0 (0.1)	0.6 (0.1)	0.06

MMSE, mini-mental state examination; LMWT, Luria memory words test; COWAT, controlled oral word association test; SST, serial sevens subtraction test.

### Electroencephalography recording and data acquisition

The registration and evaluation of EEG was carried out in accordance with the IPEG guidelines (Versavel et al., 1995; Jobert et al., 2012). All recordings were obtained in the afternoon from 3-4 p.m. EEGs were recorded for 4 min during the resting state with the subjects sitting comfortably in a chair. The subjects were asked to close their eyes and relax but to stay awake during the recording. To maintain a constant level of vigilance, an experimenter monitored the subject and the EEG traces online and verbally alerted the subject any time there were signs of behavior and/or EEG drowsiness.

The EEGs were recorded on a Nihon Kohden 4217 G EEG (Japan) using a time constant of 0.3 s. The high frequency cutoff was 45 Hz. The 16 Ag/AgCl electrodes were placed according to the international 10–20 system at the O2, O1, P4, P3, C4, C3, F4, F3, Fp2, Fp1, T6, T5, T4, T3, F8, and F7 positions. Linked ears served as the reference. The electrode impedance did not exceed 10 kΩ. During the recordings, 180 s of EEG at rest was simultaneously sampled at 256 Hz per channel and stored on a computer for further offline analysis. The EEG was reviewed visually for artifacts, which were eliminated from the subsequent analysis. After the artifacts were eliminated, 150-s segments of the resting EEG were selected for further analysis. Frequencies below 2 Hz and above 35 Hz were eliminated using digital filtering.

Recent studies emphased the importance of using narrow bands instead of broad bandwidths (e.g., the whole of the “alpha” ranges), as the standard bands may mask subfrequencies of functional importance. In this study we applied the analysis of 1-Hz alpha subbands relative power and IAPF.

The relative power (% of total EEG power) of every 1-Hz subband of the alpha bands (8–13 Hz) was calculated. Log transformations of the relative power of the various bandwidths in each derivation were calculated to compensate for data skewness, as recommended by John et al. (1980), using log [x/(1-x)], where x is the fraction of total power for each 4-s sample. The average log relative power for each frequency band was then calculated. The details of the spectral analysis procedures have been previously described (Ponomareva et al., 2017). The IAPF, the frequency at which the maximum alpha power occurs, were computed for each subject as described previously (Yaakub et al., 2020).

### Genotyping

Genomic DNA was isolated from peripheral venous blood by the standard phenol_chloroform extraction methodology or using a Qiagen kit for DNA isolation. Genotyping was performed by PCR followed by RFLP analysis. Amplification was performed according to the manufacturer’s instructions using a Tercyc DNA amplifier (DNA Technology, Moscow, Russia) and GeneAmp PCR System 9700 Thermal Cycler (Applied Biosystems).

To genotype the *APOE* gene locus, the following oligonucleotide primers were used: 5′_CGGCTGGGCGCG_GACATGGAGGA and 5′_TCGCGGGCCCCGGC_CTGGTACAC. The PCR protocol was as follows: preliminary denaturation at 95°C for 4 min; 5 cycles: 95°C for 45 s, 54°C for 25 s, and 72°C for 30 s; and 30 cycles: 95°C for 5 s, 58°C for 15 s, and 72°C for 5 s; the last stage was performed at 72°C for 3 min. PCR products were then cleaved by *Hha*I or *Bst*HHI (SibEnzyme, Novosibirsk, Russia) and restriction products were analyzed in a 7.5% polyacrylamide gel (Golenkina et al., 2010).

### fMRI imaging acquisition

In addition to all other examinations, fMRI rsFC was examined in a subgroup of 37 individuals, which included 11 men and 26 women (age range 29–79 years, mean age 54.1 + 1.8). Among these individuals, 23 were *APOE4+* non-carriers (age-range 31–71 years, mean age 55.4 + 2.0), and 14 were *APOE4+* carriers (age range 29–73 years, mean age 51.9 + 3.5) (Table 2).

**TABLE 2:** Demographic and psychometric characteristics of participants with fMRI evaluation.

	*APOE4–*	*APOE4+*	*p*
*N*	23	14	
Age, years SE	55.4 (2.0)	51.9 (3.5)	0.4
Sex m/w	8/15	3/11	0.4
Education, years (SE)	14.9 (0.1)	14.9 (0.6)	0.9
MMSE (SE)	29.8 (0.1)	29.8 (0.2)	0.9
LMWT (SE)	5.5 (0.3)	5.5 (0.4)	0.9
COWAT (SE)	51.4 (2.8)	52.4 (2.1)	0.8
SST (SE)	1.0 (0.1)	0.8 (0.2)	0.7

Abbreviations are the same as in Table 1.

Magnetic resonance imaging (MRI) studies were performed on the MAGNETOM Verio magnetic resonance tomograph (Siemens, Germany) with a 3.0T magnetic field.

Structural images were acquired using a T1-weighted MPRAGE sequence: TR = 1,900 ms, TE = 2.47 ms; FOV = 256 × 256 mm^2^; flip angle = 10°; slice thickness 1.0 mm; interslice distance 1 mm; number of slices = 176.

Functional scans were obtained at rest using T2*-weighted EPI sequence: TR = 1,500 ms, TE = 30 ms, flip angle 70°, slice thickness 2 mm, FOV = 190 mm, FoV phase 100.0%. The subjects were instructed to relax as much as possible, to lie quietly with their eyes closed (to exclude stimulation of the visual system) and not to think about anything in particular.

For analysis of rsFC signals in fMRI images we used CONN, which is a MATLAB-based open source toolbox (Functional Connectivity SPM Toolbox 2017, McGovern Institute for Brain Research, Massachusetts Institute of Technology)^1^ (Whitfield-Gabrieli and Nieto-Castanon, 2012).

CONN toolbox version 18b in conjunction with the SPM 12 software package (Wellcome Department of Cognitive Neurology, London, United Kingdom)^2^ was used to perform all preprocessing steps. Functional images were slice-time corrected, realigned (motion corrected), and coregistered to their respective T1-weighted anatomical image. Images were then normalized to the Montreal Neurological Institute (MNI) standard space and spatially smoothed with an 8-mm Gaussian filter.

Denoising methods was then applied to minimize the impact of artifactual sources of signal variability. This included band-pass filtering (0.01–0.1 Hz), scrubbing (volumes showing displacement larger than the 97th percentile were censored), regressing out of the first 10 principal components (aCompCor) calculated within the maps of white matter (five components) and cerebrospinal fluid (five components) and regressing out of 24 head motion parameters, including linear and rotational indices, their temporal derivatives and their squared values.

The CONN-18.b toolbox was used to obtain a linear measure of functional connectivity based on bivariate correlation and bivariate regression coefficients between seed areas for ROI-to-ROI analysis (Whitfield-Gabrieli and Nieto-Castanon, 2012). ROIs of the whole brain were drawn from the template provided by CONN (conn/rois/atlas.nii). For the purpose of the analysis, BOLD signal time courses were converted to normally distributed scores with Fisher’s transformation, which allows for the use of second-level general linear Model analysis.

We performed a region of interest (ROI) analysis whereby ROIs were anatomically defined using the FSL Harvard-Oxford maximum probability cortical atlas, with bilateral regions divided into left and right hemispheres (168 ROIs).

### Statistical analysis

Differences in demographic scores between the groups (*APOE4–* younger, *APOE4+* younger, *APOE4–* older, *APOE4+* older) were tested using analysis of variance (ANOVA) for continuous variables (age, education), and the Mann–Whitney *U* test for categorical variables (sex).

EEG parameters from each group were tested for normal distribution by the Wilk-Shapiro test, and in no cases were the data skewed. The significance of the differences was estimated for IAPF using repeated-measures ANOVA in the general linear model (GLM), with genotype group (*APOE4–*vs. *APOE4*+) and age (younger vs. older) as a between subjects factor, and ROI: occipital (O2, O1), parietal (P4, P3), central (C4, C3), frontal1 (F4, F3), frontal2 (Fp2, Fp1), temporal1 (T6, T5), temporal2 (T4, T3), temporal3 (F8, F7), and hemisphere (right, left) as a within- subject factor. Sex was included in the analysis as covariate.

The significance of the differences between the log-transformed alpha relative power was estimated using repeated-measures ANOVA in the GLM, with genotype group (*APOE4–* vs. *APOE4*+), and age (younger vs. older) as a between subjects factor, and bands (for the 1-Hz subbands of alpha (8–13 Hz) as a within-subject factor.

*Post-hoc* comparisons for between-subject effects and within-subject effects were analyzed using the Duncan test, and the level of significance was set to *p* < 0.05.

We compared ROI-to-ROI connectivity rsFCs between the *APOE4+* and *APOE4–* cohorts using two-tailed paired *t*-tests. The resulting statistical maps were set with *p* < 0.05 (FDR corrected).

The comparison EEG and fMRI was performed in the same participants. Correlation analysis between the EEG characteristics and age of the individuals, as well as Pearson correlation analysis of EEG characteristics and fMRI rsFC values was performed in cases of normal distribution and Spearman rank correlations were calculated in other cases. An uncorrected significance level of *p* < 0.05 was considered to indicate a tendency.

## Results

The participant groups were not significantly different in age or sex as well as psychometric characteristics for any of the datasets when assessed using one-way analyses of variance and χ^2^ tests (Tables 1, 2).

There were no differences in age, sex, education and cognitive characteristics between the *APOE4+* carriers and non-carriers in either the young or the old subgroups, or in the whole sample (*p* > 0.05). There were no significant differences in sex between the young and the old subgroups with the same *APOE* genotype.

### Comparison of electroencephalography alpha power spectrum in non-demented individuals with different *APOE* genotypes

**FIGURE 1:**
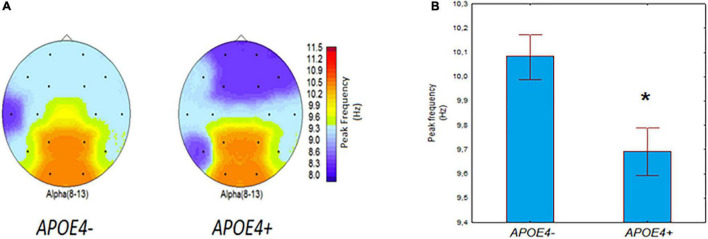
EEG individual alpha peak frequency (IAPF) in non-demented *APOE4–* and *APOE+* carriers. **(A)** Group-averaged EEG topographical plots of IAPF in individuals with *APOE4+* and *APOE4–* genotypes. **(B)** IAPF (mean and SE) in individuals with *APOE4+* and *APOE4–* genotypes. *p = 0.01; significant difference between *APOE4+* vs. *APOE4–* subjects.

The results of ANOVA revealed a significant effect of the *APOE* genotype (*APOE4+* vs. *APOE4–*) on IAPF (*F*_[1,135]_ = 6.71, *p* = 0.01). For illustrative purposes, Figure 1A maps the average IAPF in the *APOE4+* carriers and non-carriers. In the *APOE4+* carriers IAPT was reduced compared to the *APOE4+* non-carriers (Figure 1B).

**FIGURE 2:**
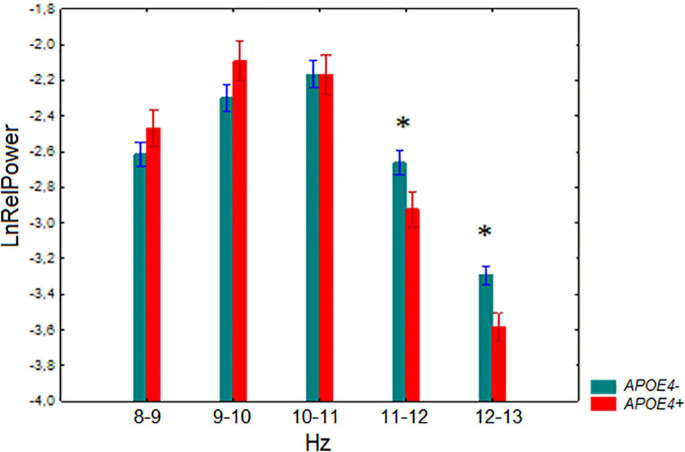
Log-transformed relative power (mean and SE) in the non-demented subjects with *APOE4+* and *APOE4–* genotypes. *p < 0.05; significant difference between *APOE4+* vs. *APOE4–* subjects of every 1-Hz subband of alpha band.

A significant interaction effect between the factors *APOE* genotype and 1-Hz alpha bands was observed *F*_[4,524]_ = 3.67, *p* = 0.006. *Post-hoc* comparison showed that the relative power of 11–12 Hz and 12–13 Hz was lower in the *APOE4+* carriers than in the non-carriers (*p* = 0.005 and *p* = 0.007 for 11–12 Hz and 12–13 Hz, respectively) (Figure 2).

**FIGURE 3:**
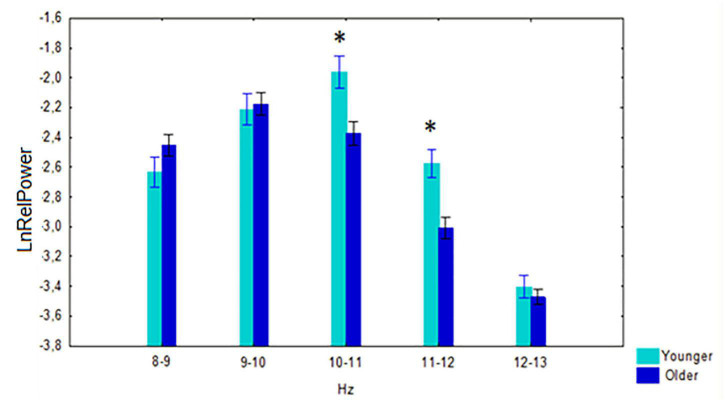
Log-transformed relative power (mean and SE) in the non-demented younger and older cohorts. *p < 0.01; significant difference between younger vs. older subjects of every 1-Hz subband of alpha band.

A significant interaction effect between the factors age and 1-Hz alpha bands was observed *F*_[4,524]_ = 5.68, *p* < 0.001 . *Post-hoc* comparison showed that the relative power of 10–11 Hz (*p* < 0.001) and 11–12 Hz (*p* < 0.001) was higher in the younger subjects than in the older (Figure 3).

**FIGURE 4:**
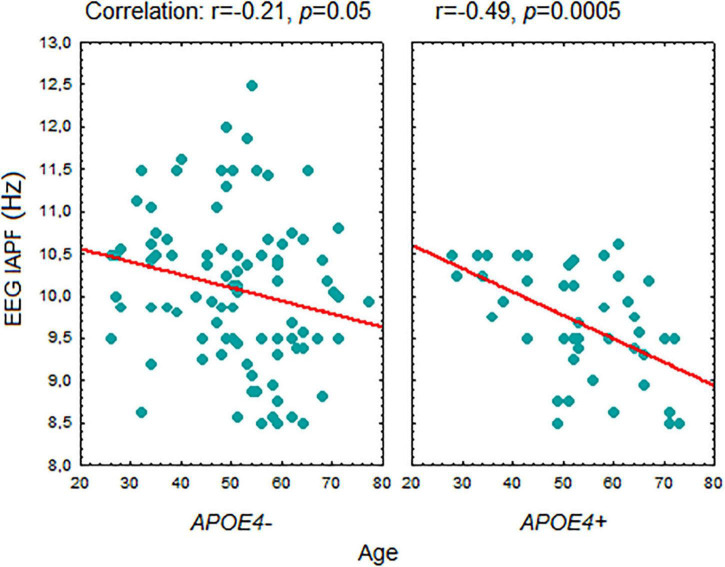
Correlation between age and EEG individual alpha peak frequency in *APOE4+* and *APOE4–* healthy subjects. Abbreviations are the same as in Figure 1.

A correlation between age and IAPF was significant in the carriers of the *APOE4+* genotype (*r* = –0.49, *p* = 0.0005). In the *APOE4+* non-carriers the correlation with age was weaker (*r* = –0.21, *p* = 0.046) (Figure 4). The difference between the correlation coefficients in *APOE4+* carriers and non-carriers assessed by the Fisher r-to-z transformation was significant (*p* = 0,043 one sided test).

### Comparison of fMRI resting-state functional connectivity in in non-demented individuals with different *APOE* genotypes

**FIGURE 5:**
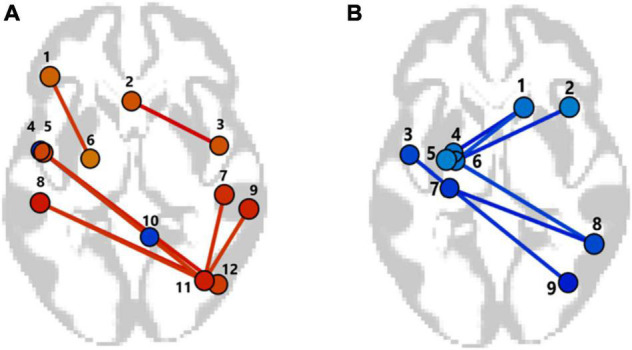
The patterns of functional links, corresponding to the fMRI resting-state functional connectivity (rsFC) differences in the non-demented *APOE4+* carriers and non-carriers (*p*-FDR < 0.05). **(A)** Between groups contrast *APOE4+* > *APOE*–. Only the networks with significant positive rsFC values in *APOE4+* carriers or in non-carriers (*p*-FDR < 0.05) are presented. 1—Lateral PreFrontal Cortex left, LPFC l; 2—Subcallosal Cortex SubCalC; 3—Inferior Temporal Gyrus right, anterior division, aITG r; 4—Inferior Temporal Gyrus left, anterior division, aITG l; 5—Planum Polare left, PP l; 6—Anterior Parahippocampal Gyrus left, aPaHC l; 7—Parietal Operculum right, PO r; 8—Parietal Operculum left, PO l; 9—Salience Supramagrinal right, Salience SMG r; 10—Cerebellum9 right, Cereb9 r; 11—Visual Laterale right, VisLat r; 12—Lateral Occipital Cortex right iLOC r. **(B)** Between groups contrast *APOE4–* < *APOE4+.* Only the networks with significant negative rsFC values in *APOE4–* or *APOE+* carriers (*p*-FDR < 0.05) are presented. 1—Superior Frontal Gyrus right, SFG r; 2—Middle Frontal Gyrus right, MFG, r; 3—Planum Polare left, PP l; 4—Amygdala; 5—Dorsal Attentional Network, Dor Att Net; 6—Anterior Parahippocampal Gyrus left, aPaHC l; 7—Hippocampus left; 8—Posterior Parietal Cortex right, PPC; 9—Lateral Occipital Cortex right, iLOC r.

The ROI to ROI significant fMRI rsFC differences between the *APOE4+* carriers and non-carriers are presented in Figures 5A,B and in Tables 3, 4. The *APOE4+* carriers had higher fMRI positive rsFC of the interhemispheric regions of the frontoparietal, lateral visual and salience networks than the *APOE4–* individuals (Figure 5A and Table 3). Compared to *APOE4+* carriers, *APOE4–* individuals had stronger fMRI positive rsFC only in the network between the right cerebellum and the left inferior temporal gyrus.

**TABLE 3:** ROI-to-ROI resting state functional connectivity (rsFC) according to fMRI of the *APOE4+* and non-carriers.

Analysis unit	Groups	Beta	T	*p*-unc	*p*-FDR
aITG r—SubCalC	*APOE4+ vs. APOE4–*	0.22	4.68	0.00005	0.0077
	*APOE4+*	0.21	5.38	0.0001	0.0015
	*APOE4–*	–0.01	–0.25	0.8	0.88
iLOC r—PO l	*APOE4+ vs. APOE4–*	0.25	4.5	0.0001	0.0109
	*APOE4+*	0.26	5.9	0.00005	0.00076
	*APOE4–*	0.00	0.11	0.92	0.93
iLOC r—PP l	*APOE4+ vs. APOE4–*	0.21	4.32	0.0001	0.0109
	*APOE4+*	0.14	3.4	0.00.48	0.024
	*APOE4–*	–0.07	–2.5	0.02	0.08
VisLat r—PO r	*APOE4+ vs. APOE4–*	0.26	3.98	0.0004	0.0198
	*APOE4+*	0.23	7.49	0.000005	0.00008
	*APOE 4–*	–0.03	–0.54	0.6	0.69
VisLat r—PO l	*APOE4+ vs. APOE4–*	0.29	3.97	0.0004	0.0198
	*APOE4+*	0.2	5.	0.0002	0.003
	*APOE4–*	–0.1	–1.8	0.09	0.19
VisLat r—Salience SMG r	*APOE4+ vs. APOE4–*	0.28	3.87	0.0005	0.02
	*APOE4+*	0.24	7.1	0.000008	0.0001
	*APOE4–*	–0.04	0.08	0.43	0.57
aPaHC l—LPFC l	*APOE4+ vs. APOE4–*	0.2	3.83	0.0005	0.029
	*APOE4+*	0.13	4.0	0.00012	0.013
	*APOE4–*	–0.07	–1.9	0.07	0.15
Cereb9-aITG l	*APOE4+ vs. APOE4–*	–0.24	–4.17	0.0002	0.03
	*APOE4+*	–0.11	–2.06	0.06	0.165
	*APOE4–*	0.13	4.28	0.000363	0.0021

Between groups contrast *APOE4+ > APOE-*. Only the networks with significant positive rsFC values in *APOE4+* carriers or non-carriers are presented (*p*-FDR < 0.05). In all these networks, except one (Cereb9-aITG l), significant positive rsFC values were observed in *APOE4+* carriers. The data correspond to Figure 5A. Abbreviations are the same as in Figure 5.

**TABLE 4:** ROI-to-ROI resting-state functional connectivity (rsFC) according to fMRI of the *APOE4+* and non-carriers.

Analysis unit	Groups	Beta	*T*	*p*-unc	*p*-FDR
VisLat r—PP l	*APOE4- vs. APOE4+*	–0.24	–3.93	0.0004	0.0198
	*APOE4+*	0.11	2.47	0.028	0.1
	*APOE4–*	–0.13	–3.29	0.0036	0.02
SFG r—Amygdala l	*APOE4- vs. APOE4+*	–0.18	–4.24	0.0002	0.028
	*APOE4+*	0.01	0.57	0.58	0.66
	*APOE4–*	–0.17	–5.39	0.00003	0.0003
aPaHC l—MidFG r	*APOE4- vs. APOE4+*	–0.17	–4.05	0.0003	0.0291
	*APOE4+*	–0.02	–0.68	0.51	0.65
	*APOE4–*	–0.2	–7.23	0.000001	0.00001
aPaHC l—DorAtt Net FEF l	*APOE4- vs. APOE4+*	–0.17	–3.96	0.0004	0.03
	*APOE4+*	0.06	1.64	0.12	0.26
	*APOE4–*	–0.11	–4.29	0.0004	0.0027
aPaHC l—PPC r	*APOE4- vs. APOE4+*	–0.18	–3.68	0.0008	0.034
	*APOE4+*	0.03	0.63	0.54	0.67
	*APOE4–*	–0.15	–5.45	0.000025	0.0003
aPaHC l—SFG r	*APOE4- vs. APOE4+*	–0.2	–3.59	0.0011	0.035
	*APOE4+*	–0.04	–1.08	0.299	0.49
	*APOE4–*	–0.24	–6.59	0.000002	0.00003
Hippocampus l—PPC r—	*APOE4+ vs. APOE4–*	–0.24	–4.14	0.0002	0.037
	*APOE4+*	0.00	–0.05	0.96	0.97
	*APOE4–*	–0.25	–6.37	0.000003	0.000045

Brain networks with significant negative differences in rsFC between *APOE4-* vs. *APOE4+*. Only the networks with significant negative rsFC values in *APOE4+* carriers and non-carriers are presented (*p*-FDR < 0.05). In all these networks significant negative rsFC values were observed in *APOE4+* non-carriers. The data correspond to Figure 5B. Abbreviations are the same as in Figure 5.

In contrast, in the *APOE4+* non-carriers the negative rsFC in the networks of the left hippocampus and the right posterior parietal cortex (PPC), as well as the negative rsFC between the left parahippocampal gyrus and the right superior frontal gyrus was stronger than in the *APOE4+* carriers. In the *APOE4+* non-carriers the negative rsFC in these networks was significant, while for the *APOE4+* carriers rsFC of these networks was non-significant (Figure 5B and Table 4).

### Electroencephalography alpha correlates of fMRI networks in individuals with different the *APOE* genotypes

In the subgroup of subjects with fMRI examination the main effect of the *APOE* genotype on IAPT was similar to that, found in the whole sample (*F*_[1,28]_ = 7.64, *p* = 0.01).

**FIGURE 6:**
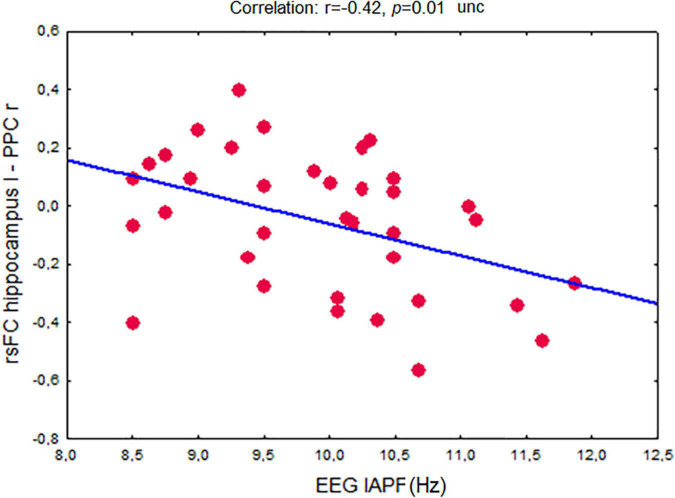
Association between fMRI-derived resting-state functional connectivity in the network between the left hippocampus and the right posterior parietal cortex and EEG individual alpha peak frequency in non-demented adults. Abbreviations are the same as in Figure 1.

EEG IAPF showed a significant inverse correlation between rsFC of the left hippocampus and the right PPC (*r* = –0.42, *p* = 0.01 uncorrected) (Figure 6).

## Discussion

The results of this study show that the presence of the *APOE4+* genotype in non-demented adults is associated with IAPF reduction and lower relative power of the high-frequency 11–13 Hz alpha subbands, and the effect of *APOE* genotype on alpha rhythm characteristics was increased in an age-dependent manner.

In addition, we found that, compared to non-carriers of the *APOE4+* genotype, the *APOE4+* carriers had higher positive rsFC of interhemispheric regions of the frontoparietal, visual lateral and salience networks. In contrast, in the *APOE4+* non-carriers the negative rsFC of the networks between the left hippocampus and right PPC as well as of the networks between the parahippocampal gyrus and right PPC was significant, while for the *APOE+* carriers the rsFC of these networks was not found to be significant.

Across the whole group of participants EEG IAPF showed a significant inverse relationship with the fMRI rsFC of the left hippocampus and the right PPC network. An increase of IAPF was associated with a decrease in rsFC.

### Electroencephalography alpha power spectrum in non-demented individuals with different *APOE* genotypes

Previous studies consistently demonstrated an association of EEG slowing with *APOE4+* genotype in AD and MCI patients (Lehtovirta et al., 2000; Babiloni et al., 2006; Ponomareva et al., 2008; Cuesta et al., 2015). Compared to *APOE4–* patients, spontaneous EEG of *APOE4+* patients with MCI and AD demonstrated higher spectral power of delta and theta frequencies and lower alpha-activity. The effects of *APOE* on alpha activity in the brain were suggested to be due to alterations in cholinergic pathways. Recent MEG studies found that in healthy *APOE4+* carriers older than 48 years of age the IAPF was shifted toward lower frequencies (de Frutos-Lucas et al., 2018, 2020). In the present study, we applied EEG analysis to investigate the association between the *APOE* genotype and IAPF in non-demented adults over a wider age range. We found that this association was age-dependent.

The characteristics of IAPF and the relative power of alpha subbands are interrelated with each other and affected by synapse loss. Aging is characterized by alpha rhythm slowing, which occurs even in the absence of brain disease. The present study shows that the age-related decrease of EEG IAPF and relative power of 11-13 Hz alpha subbands are more pronounced in carriers of the *APOE4+* genotype than in carriers of the *APOE4–* genotype.

### fMRI resting-state functional connectivity in non-demented individuals with different *APOE* genotypes

The results of the present study showed significant differences in the fMRI rsFC parameters of the brain networks in *APOE+* carriers and non-carriers. Functional MRI studies have demonstrated that the brain is organized into distributed anticorrelated networks (Fox et al., 2005). Task-positive networks, including frontoparietal, visual lateral and salience networks, are involved in cognitive control and attention modulation. These networks become more active during external goal-directed tasks. In contrast, the DMN, which plays an essential role in memory function, demonstrates a decrease in activity when the individual is engaged in external goal-directed tasks (Sheline and Raichle, 2013). In healthy adults, resting state connectivity between task positive and task negative networks is anticorrelated, i.e., shows a negative correlation. The results of the present study indicate that, compared to non-carriers, the *APOE4+* carriers had higher positive rsFC in interhemispheric regions of the frontoparietal, visual lateral and salience networks. In contrast, the *APOE4+* non-carriers demonstrated significant negative rsFC for networks between the left hippocampus and right PPC as well as for networks between the parahippocampal gyrus and right PPC, while in the *APOE4+* carriers the rsFC of these networks was not significant.

The majority of studies have found that, compared to healthy age-matched controls, AD and MCI patients demonstrated decreased functional connectivity in task-positive networks, including dorsal attention and salience networks, as well as the DMN (Sheline and Raichle, 2013; Brier et al., 2014a; Puttaert et al., 2020). Some studies reported that, compared to non-carriers, clinically healthy *APOE4+* carriers demonstrated decreased rsFC in the DMN and medial temporal lobe (Yan et al., 2015; Lu et al., 2017; Su et al., 2017; Pietzuch et al., 2021), but other studies have found the opposite alterations (Filippini et al., 2011; Wu et al., 2016; Kagerer et al., 2020). Local Aβ and tau accumulation in the brain was found to be associated with functional connectivity in brain networks, and a positive interaction was observed for the *APOE4* genotype and functional connectivity with brain regions characterized by increased local Aβ and tau accumulation (Quevenco et al., 2020).

### Electroencephalography alpha correlates of fMRI networks in individuals with different *APOE* genotypes

In the present study we found that a decrease in EEG IAPF was linked to decrease of inhibitory rsFC of networks between the hippocampus and the posterior parietal cortex. This finding supports the notion that alpha rhythm vulnerability in *APOE4+* carriers reflects a key feature of AD pathophysiology.

Alpha rhythm generation results from cortico-thalamo-cortical, and intracortical circuits (Li et al., 2020). However compelling evidence shows that hippocampal networks also provide a modulating effect on alpha rhythm (Knaut et al., 2019; Martín-Buro et al., 2020). In the group of MCI patients and normal controls the decrease in alpha peak frequency correlated with hippocampal volume (Garcés et al., 2013). AD patients demonstrated a decreased negative association of hippocampal fMRI rsFC with alpha power (Brueggen et al., 2017).

The hippocampal formation plays an essential role in learning and memory function (Jiménez-Balado and Eich, 2021). The hippocampus was shown to be one of the first regions affected in aMCI and AD. The volume of the hippocampus and functional connectivity in hippocampal networks are disrupted in both AD and MCI carriers of the *APOE4+* genotype and, to a lesser extent, in clinically healthy elderly *APOE4+* carriers (Brier et al., 2014b; Schultz et al., 2017; Puttaert et al., 2020).

An experimental study in *APOE4+* mice demonstrated complementary alterations of brain electrophysiology and fMRI rsFC of brain networks that precede accumulation of Aβ and tau pathology in the brain (Nuriel et al., 2017). Electrophysiological analysis of aged *APOE4+* mice revealed neural hyperactivity and increased duration of spontaneous synchronized events in the entorhinal cortex, which is the main interface between the hippocampus and the neocortex (Schultz et al., 2015). This decrease in inhibition in *APOE4+* mice is caused by a reduced responsiveness of excitatory neurons to GABAergic input. The authors hypothesized that this *APOE4+* -related shift in the excitation/inhibition balance can eventually result in increased amyloid deposition, and therefore an increased risk of AD development.

We have previously found that in healthy adults under a hyperventilation test, the presence of the *APOE4+* genotype is associated with the manifestation of synchronous high-amplitude delta-theta activity and sharp-waves. Such phenomena occur in the EEG of *APOE4+* in thalamo-cortical synchronizing systems as well as from cerebral hyperexcitability (Ponomareva et al., 2008). *APOE4+* carriers demonstrate a higher incidence of epilepsy (Lamoureux et al., 2021). Recent studies have found IAPF slowing in patients with epilepsy and in their asymptomatic relatives. These findings suggest the possibility of the existence of genetic factors that contribute to the reduction of IAPF and to the alterations of the brain networks related to alpha oscillation (Yaakub et al., 2020).

Loss of tonic inhibition manifests in AD patients as hypersynchrony, leading to epilepsy and aberrant activation of cortical and hippocampal networks (Palop and Mucke, 2009). Inhibitory deficits of these networks contribute to learning and memory impairments (Koelewijn et al., 2019).

The limitations of the present study include a relatively small number of participants, who underwent fMRI examination. This suggests that the results on the association of IAPF and rsFC are preliminary. Nevertheless, the finding on the interdependence of the EEG and fMRI characteristics can contribute to an understanding of the underlying mechanisms behind alpha rhythm slowing in *APOE4+* carriers.

Another limitation is the non-simultaneous registration of EEG and fMRI. However, as in the resting state of healthy subjects these characteristics are relatively stable, and separate EEG and fMRI recordings across experimental sessions are sufficient (Scrivener, 2021).

Taken together, the results of our study show for the first time that, in clinically healthy subjects, the association of *APOE4+* genotype with alpha rhythm slowing is age-dependent. According to fMRI results, this genotype is associated with rsFC alterations of large-scale brain networks. These alterations are expressed by an increase in rsFC in networks with positive connectivity and by a decrease in rsFC in networks with negative connectivity. Alpha rhythm slowing was associated with the dysfunction of hippocampal networks. All abnormalities mentioned above point to the conclusion that the *APOE4+* genotype carriers demonstrate dysfunction of brain networks with a prevalence of alterations of inhibitory processes.

## Data availability statement

The original contributions presented in this study are included in the article/supplementary material, further inquiries can be directed to the corresponding authors.

## Ethics statement

The studies involving human participants were reviewed and approved by Ethics Committee of Research Center of Neurology, Moscow, Russia. The patients/participants provided their written informed consent to participate in this study.

## Author contributions

NP, ER, and SI contributed to the conceptualization of this manuscript and designed the research. NP, TA, MP, RK, MK, EPK, DM, and EVK performed the research. NP, VF, AM, ER, and RK contributed to the data analysis. NP wrote the manuscript. All authors contributed to the article and approved the submitted version.

## Footnotes

1
http://www.nitric.org/projects/conn

2
www.fil.ion.ucl.ac.uk/spm
